# The long-lasting impact of male-based research on female physiology—also true for slow wave sleep

**DOI:** 10.1093/sleep/zsaf181

**Published:** 2025-07-07

**Authors:** Monika Haack

**Affiliations:** Department of Neurology, Beth Israel Deaconess Medical Center, Boston, MA, United States; Harvard Medical School, Boston, MA, United States

The study by Davidson et al. [1] reports on how standard American Academy of Sleep Medicine (AASM)-based slow wave sleep (SWS) visual scoring criteria, characterized by a fixed 75 μV amplitude criteria in addition to a frequency criteria of 0.5–2 Hz, affects age- and sex-related trends in comparison to an automated scoring method using data-driven amplitude and frequency criteria. This comparison of visual and automated SWS labeling approaches was a large undertaking and tested in a cohort of almost 3000 participants from the Sleep Heart Health Study. The fundamental finding is that the fixed 75 μV amplitude threshold criteria is creating artifactual, rather than physiological SWS trends for sex across aging. Specifically, applying classical SWS scoring criteria leads to a decrease of SWS between the ages of 40 and 80 years in males only, while SWS in females increases across age. However, using automated data-driven amplitude and frequency criteria, males show the same trend as for visual scoring i.e. a decrease in SWS across aging, but in females, the visually observed SWS increase across aging could not be verified with the automated data-driven analysis. Thus, both SWS scoring methods produce similar trends in males, but inconsistent trends in females. The reason behind these different outcomes may relate to the participant sample that was used to develop SWS scoring criteria, as we know them today: They were developed using EEG recordings from a small sample of mostly young male participants between the ages of 20 and 30 years, as outlined by Davidson et al. This reflects the long history of male-dominated research, starting in 1977 when the US Food and Drug Administration (FDA) excluded females of childbearing potential from clinical studies and drug trials, mainly driven by certain drugs causing potential birth defects. This exclusion also happened in basic and translational science, though exclusion here was also driven by the inconvenience of the presumably greater variability of female physiology due to changing hormonal patterns across the menstrual cycle and across different life phases. This exclusion resulted in taking drugs off the market or adjust drug dosing, because of unexpected adverse drug reactions in females—a result of the insufficient enrollment of females in clinical and preclinical drug trial [2]. This practice of not enrolling females or not adequately analyzing sex differences ended finally in 2016, when the National Institutes of Health NIH)-enforced policies to consider sex as a biological variable [3, 4]. There is increasing awareness now that sex can be a powerful factor that differentially influence many biological, physiological, and psychological aspects. Still, as of to date, the sleep field continues to use SWS scoring criteria developed in the sixties in a small group of mainly young males, and applies them to both sexes, from young adults to old age.

How has this practice influenced, and potentially distorted, our scientific-based knowledge on sex differences in SWS across aging and the role of SWS for biological processes? A review by Carrier et al. [5] on age-related changes in the sleep–wake cycle in women and men reports on sex differences in numerous sleep aspects across the aging process, among them a more rapid decline of SWS in men than women found in a few studies. That has led to the suggestion that sleep in men may age more rapidly than sleep in women [6]. In light of the findings by Davidson, one may also suggest that this finding is an artifact rather than a physiological fact. SWS has been associated with restorative and neuroplasticity processes, including aspects of the innate and adaptive immune system and memory consolidation [7]. Dysregulations of these processes are evident in various chronic diseases, including autoimmune diseases, chronic pain conditions, or dementia, almost all of which show a pronounced sexual dimorphism, with females overwhelmingly more affected in most chronic diseases. Having SWS scoring criteria that accurately reflect physiological processes in both sexes, is one important condition that increases rigor in the investigation of a potentially sex-differential role of SWS in the development, severity, or persistence in various clinical conditions, and may offer insights toward sex-tailored therapeutics aimed at manipulating SWS characteristics.

Davidson et al. highlight the need for the AASM to update their SWS scoring criteria to ensure that they perform equally well in both sexes. An adoption of slow wave (SW) amplitude criteria by age and sex has been recommended before, and many in the sleep field have probably heard that the 75 μV amplitude criteria is too high for older adults. The findings by Davidson et al. may hopefully be a final push toward facilitating the review and revision of current standard SWS scoring criteria. A data-driven approach, such as the one used by Davidson et al., can guide such revision towards age- and sex-adapted SW amplitude criteria. Visual scoring will likely be around for the near future, so age- and sex-based SWS adjustments are still important to increase rigor in the studies to come that involve visually based SWS criteria, and females. Beside the long-standing use of visual scoring criteria in research, there are rapid advances in automated AI/machine learning-based approaches for the detection and characterization of SWS, with exciting discoveries on SWS aspects for human health and the potential to therapeutically target SWS characteristics [7, 8]. While these are exciting discoveries, we need to be aware that automated SWS analysis approaches may not automatically perform equally well for both sexes, because they may not necessarily be completely independent from visually labeled N2 and N3 epochs. As outlined in Davidson et al., visually labeled N2 and N3 epochs have been used for some calibration processes in their automated data-driven method. Though Davidson minimized a possible effect using sensitivity analysis, it is important to be aware that criteria developed and research performed on males only do not pervade our scientific understanding of female physiology.
